# Hiding in plain sight: The discovery of complete genomes of 11 hypothetical spindle‐shaped viruses that putatively infect mesophilic ammonia‐oxidizing archaea

**DOI:** 10.1111/1758-2229.13230

**Published:** 2024-01-23

**Authors:** Yimin Ni, Tianqi Xu, Shuling Yan, Lanming Chen, Yongjie Wang

**Affiliations:** ^1^ College of Food Science and Technology Shanghai Ocean University Shanghai China; ^2^ Entwicklungsgenetik und Zellbiologie der Tiere Philipps‐Universität Marburg Marburg Germany; ^3^ Laboratory of Quality and Safety Risk Assessment for Aquatic Products on Storage and Preservation (Shanghai) Ministry of Agriculture Shanghai China; ^4^ Laboratory for Marine Biology and Biotechnology Qingdao National Laboratory for Marine Science and Technology Qingdao China

## Abstract

The genome of a putative *Nitrosopumilaceae* virus with a hypothetical spindle‐shaped particle morphology was identified in the Yangshan Harbour metavirome from the East China Sea through protein similarity comparison and structure analysis. This discovery was accompanied by a set of 10 geographically dispersed close relatives found in the environmental virus datasets from typical locations of ammonia‐oxidizing archaeon distribution. Its host prediction was supported by iPHoP prediction and protein sequence similarity. The structure of the predicted major capsid protein, together with the overall *N*‐glycosylation site, the transmembrane helices prediction, the hydrophilicity profile, and the docking simulation of the major capsid proteins, indicate that these viruses resemble spindle‐shaped viruses. It suggests a similarly assembled structure and, consequently, a possibly spindle‐shaped morphology of these newly discovered archaeal viruses.

## Graphical abstract

A putative *Nitrosopumilaceae* virus with a spindle‐shaped morphology was identified in the Yangshan Harbour metavirome. Its potential major capsid protein was annotated manually through structural comparison, *N*‐glycosylation site recognition, hydrophilicity prediction, transmembrane helices prediction, and docking simulation. Its host prediction was supported by iPHoP prediction and protein sequence similarity. Its 10 close relatives were found in geographically diverse locations of typical ammonia‐oxidizing archaea distribution, representing a new branch of archaeal viruses of putatively spindle‐shaped morphology and global distribution.

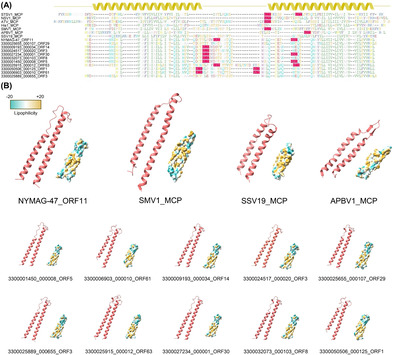

## INTRODUCTION

Archaeal viruses remain relatively unexplored compared to bacterial viruses within the broader prokaryotic virosphere, and their morphology exhibits considerable uniqueness and diversity (Krupovic et al., 2018). Currently, only 135 archaeal viruses have been documented by the International Committee on Taxonomy of Viruses (ICTV), whereas there are 5077 known bacterial viruses (Zerbini et al., 2023). Yangshan Harbour, known for its rich biodiversity (Wang et al., 2018; Wolf et al., 2020; Wu et al., 2020; Zhou et al., 2023), serves as an abundant repository of archaeal organisms. Amongst these, *Nitrososphaerota* (*Thaumarchaeota* [Oren & Garrity, 2021] was its dominant phylum (Zhou et al., 2023), which consists mostly of mesophilic organisms involved in various stages of nitrification (Reji et al., 2021). The *Nitrosopumilus* spindle‐shaped virus 1 (NSV1) was isolated from the marine ammonia‐oxidizing *Nitrosopumilus* strain SW and possesses a linear double‐stranded DNA genome of approximately 28 kbp (Kim et al., 2019). It was the first virus isolated for ammonia‐oxidizing archaea (AOA) and stands as the sole member of the family *Thaspiviridae* (Kim et al., 2021). Despite numerous reports on the discovery of spindle‐shaped archaeal viruses, which have been claimed to be the most commonly observed morphology amongst the three archaeal phyla (Baquero et al., 2020) and exclusive to archaeal viruses, no such virus with a mesophilic host had been previously found in the East China Sea.

In this study, the genome of a putative *Nitrosopumilaceae* archaeal virus with a hypothesized spindle‐shaped morphology was identified in the Yangshan Harbour metavirome from the East China Sea. This discovery was made through sequence homology comparison and structural analysis of the major capsid protein (MCP). Additionally, 10 closely related viruses were identified in the IMG/VR v4 UViG environmental dataset. These findings provide valuable insights into the diversity and distribution of archaeal spindle‐shaped viruses.

## EXPERIMENTAL PROCEDURES

### Metaviromic dataset

The metavirome dataset from Yangshan Harbour used in this study was obtained from our previous research conducted under Bioproject PRJNA610033 (Wolf et al., 2020). The samples were collected as 0.2 μm filtrates and concentrated using a 50 kDa ultrafiltration. The metaviromic sequences were subjected to quality control measures and assembled following the same procedures as described in our previous publication (Zhou et al., 2023).

### Identification of archaeal virus genomes

VIBRANT (Kieft et al., 2020) and DeepVirFinder (Ren et al., 2020) were utilized to filter viral sequences from the assembled contigs derived from the Yangshan Harbour metaviromic datasets described above. The parameter ‘‐virome’ was applied in VIBRANT, while DeepVirFinder collected sequences with a *p*‐value below 0.01. The initial viral sequence set was generated by combining the outputs from both tools. CheckV (Nayfach et al., 2021) was then employed to assess the completeness of the viral genome identified, and only complete genomes were subject to further analysis. Reads were matched to the assembled complete genome with bowtie2 (Langmead & Salzberg, 2012). The Yangshan Harbour metaviromic assembled genome identified in this study was named NYMAG‐47.

Viral hosts were originally predicted by using iPHoP (Roux et al., 2023). Additionally, tRNA gene matching and CRISPR spacer–protospacer searching methods, as described in our work (Zhou et al., 2023), were also employed for host identification. Bacterial and archaeal CRISPR spacers were collected from the CRISPR Spacer Database (Dion et al., 2021) and CRISPRCasdb (Couvin et al., 2018) for comparison, with both coverage and identity reaching above 95% considered a positive match.

### Relatives of NYMAG‐47 in IMG/VR v4

Open reading frames (ORFs) of NYMAG‐47 were predicted with Prodigal (Hyatt et al., 2010) with default parameters (translation table 11). All of the extracted protein sequences from NYMAG‐47 were aligned with DIAMOND (e‐value cutoff 1e‐5) (Buchfink et al., 2015) against the proteins of IMG/VR v4 high‐confidence database of Uncultivated Viral Genomes (UViGs) (Camargo, Nayfach, et al., 2023) and ICTV sanctioned genomes (VMR_21‐221122_MSL37) in search for related viruses in public databases. The top five and three hits for each protein sequence from the respective databases were retained, and the original sequences of the top hits for each alignment were extracted for further analysis. vConTACT2 (Bin Jang et al., 2019) was utilized for genome clustering with the cluster model command line: —pcs‐mode MCL—vcs‐mode ClusterONE. Only complete genomic sequences that were clustered with NYMAG‐47 or were identified as outliers of NYMAG‐47 were recruited and subjected to further analysis.

### Genome annotation

Tools of Cenote‐Taker 2 (Tisza et al., 2021), DRAMv (Shaffer et al., 2020), VIBRANT (−virome), and geNomad (Camargo, Roux et al., 2023) were used for genome annotation and cross‐referencing with default parameters. The HHpred online server was used for HHpred and HMM searches (Söding, 2005; Zimmermann et al., 2018). MAFFT was used for the alignment of protein sequences with default parameters (Katoh et al., 2009). The tool of trimAI was applied to aligned sequences for alignment optimization (Capella‐Gutiérrez et al., 2009). Protein cluster comparison was analysed and visualized by clinker (alignment similarity threshold of 0.30) (Gilchrist & Chooi, 2021). Alignment of protein sequences with the nr database was completed with local BLASTp (Johnson et al., 2008).

### Protein structure prediction and comparison

Protein structures were predicted with local Colabfold (Mirdita et al., 2022), compared with the Dali online server (Holm & Sander, 1995), and visualized with ChimeraX (Pettersen et al., 2021). A Dali alignment Z score of over 2 was considered a significant match (Holm et al., 2006). Local alignment during visualization was completed with Matchmaker in the ChimeraX software. The *Sulfolobus* spindle‐shaped virus 19 VP1, VP4, C131, and B210 structures were collected from PDB (https://www.rcsb.org/) (Berman et al., 2000) under the number 7XDI, and the MCPs of *Sulfolobus* monocaudavirus 1 (SMV1) and *Aeropyrum pernix* bacilliform virus 1 (APBV1) were collected under 7RO2 and 5OXE, respectively. PROMALS3D was implemented (Pei & Grishin, 2014) to align the secondary structure of MCPs. The *N*‐Glycosylation site was identified with NetNGlyc 1.0 (Gupta & Brunak, 2002), with a potential value threshold of 0.35. TMHMM 2.0 was used to predict the transmembrane‐helix‐like distribution of the proteins and hydrophobic domains (Krogh et al., 2001). The HDOCK online server (Yan et al., 2020) was utilized for MCP docking, with an empirical confidence score of 0.7 for two proteins that are likely to bind. Since the simplicity of the protein structure provides multiple configurations of similar confidence, the highest‐ranked model showing equidirectional interaction between the MCPs was chosen.

### Phylogenetic tree

MCP sequences were aligned with MAFFT (Katoh et al., 2009). Maximum likelihood phylogenetic trees were generated by IQ‐TREE with auto substitution model (Nguyen et al., 2015) and visualized with iTOL (Letunic & Bork, 2021), and the best substitution model for the aligned MCP sequences was VT determined by IQ‐TREE ModelFinder. The ViPTree online server was applied to the full genome proteomic tree (Nishimura, Yoshida, et al., 2017). A genus‐level presumption was given based on the empirical value (S_g_) proposed for a genus‐level correlation of 0.07 to 0.2 (Nishimura, Watai, et al., 2017).

### Amino acid and di‐/tetra‐nucleotide usage frequency

Reference genomes of the major microbial components in YSH (Wang et al., 2015; Zhou et al., 2023) were collected from GenBank. The accession numbers and names of the genomes collected are provided in Tables S5‐S7. For the sake of brevity and statistical accuracy, genomes of the following taxonomic groups were collected if they met the criteria of being longer than 1 Mb: *Proteobacteria*, *Acidobacteria*, *Acidimicrobiia*, *Planctomycetes*, *Gemmatimonadetes*, *Nitrospirota*, *Acidimicrobiia*, and *Chloroflexi*, and archaeal members including *Euryarchaeota*, *Nanoarchaeota* (<1 Mb), and *Crenarchaeota*. Additionally, only genomes of *Gammaproteobacteria* with lengths longer than 5 Mb were included. Genomes were translated with Prodigal. The amino acid and di‐/tetra‐nucleotide usage frequencies were calculated with the script we wrote (https://github.com/Jeffery-Ni/AANuVis). UMAP was deployed for data dimensionality reduction and visualization (metric: euclidian).

## RESULTS AND DISCUSSION

### Genomic features of NYMAG‐47 and its 10 relatives

A complete genome of 37 kbp (NYMAG‐47) was extracted from the YSH metavirome data through VIBRANT and DeepVirFinder, with an RPKM (reads per kilobase of genome per million reads mapped) of 48.8 in this dataset. Compared with the IMG/VR v4 UViGs recruited with vConTACT2, NYMAG‐47 was the outlier of the viral cluster comprising 10 other complete genomes (27–38 kbp).

Through geNomad, NYMAG‐47 was found to have five genes taxonomically related to the class *Caudoviricetes* of the kingdom *Heunggongvirae* and one gene taxonomically related to the kingdom *Bamfordvirae* (Table S3). However, structural genes of either head‐tailed viruses or *Bamfordvirae* were not found. Similar results were observed for the 10 UViGs recruited, which also had genes related to *Caudoviricetes* but lacked annotated structural genes. Particularly, no recognizable MCP was identified in the genomes of NYMAG‐47 and the 10 UViGs using various tools such as Cenote‐Taker 2, DRAMv, VIBRANT, and geNomad. Noticeably, during marker gene detection with geNomad, as control, the genome of NSV1 was also subjected to the same analysis, with five of the genes identified in total, three *Caudoviricetes* marker genes recognized, and two other genes with no taxonomic reference, reminiscent of the results NYMAG‐47 yielded. Collectively, these data indicate that geNomad v1.5.1 cannot currently identify the marker genes of NSV1 yet, and thus, the NYMAG‐47 and its relatives as well.

### MCP and virion morphotype of NYMAG‐47 and its 10 relatives

**FIGURE 1:**
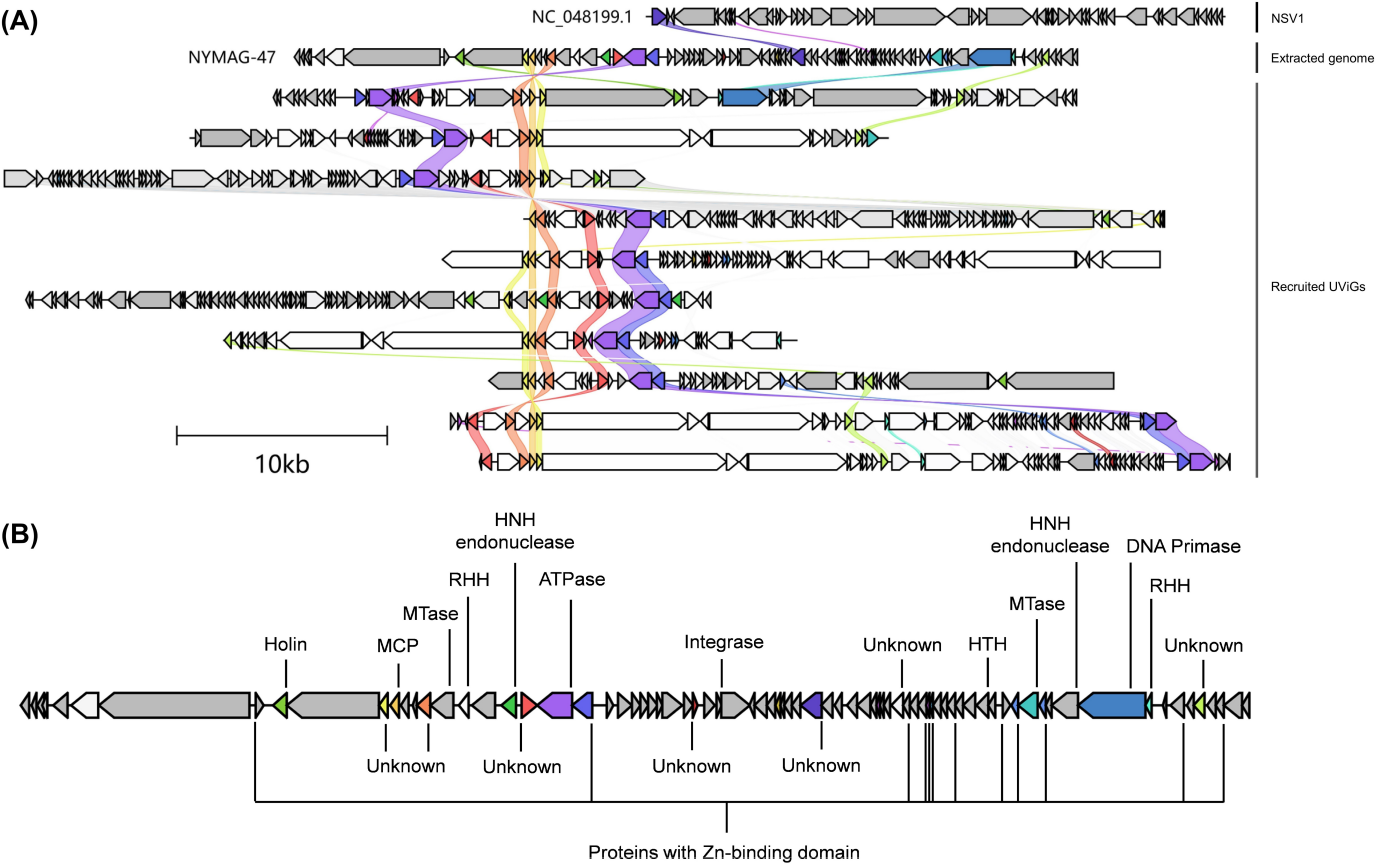
Proteins producing identity shared between NYMAG‐47 recruited genomes and NSV1 included for having putatively similar hosts. (**A**) The identity of proteins shared amongst all genomes besides NSV1 is aligned and coloured to differentiate different protein functions. Arrow orientation on the genomes indicates the ORF translation direction. Genomes are positionally aligned by the putative MCP of NYMAG‐47. (**B**) Genomic map of NYMAG‐47. Functions predicted by the conserved proteins in NYMAG‐47 are labelled, and some functions similar to that of the genes present in the NSV1 are also labelled. MCP, major capsid protein; MTase: DNA methyltransferase; RHH: ribbon‐helix–helix; HTH: helix‐turn‐helix.

**FIGURE 2:**
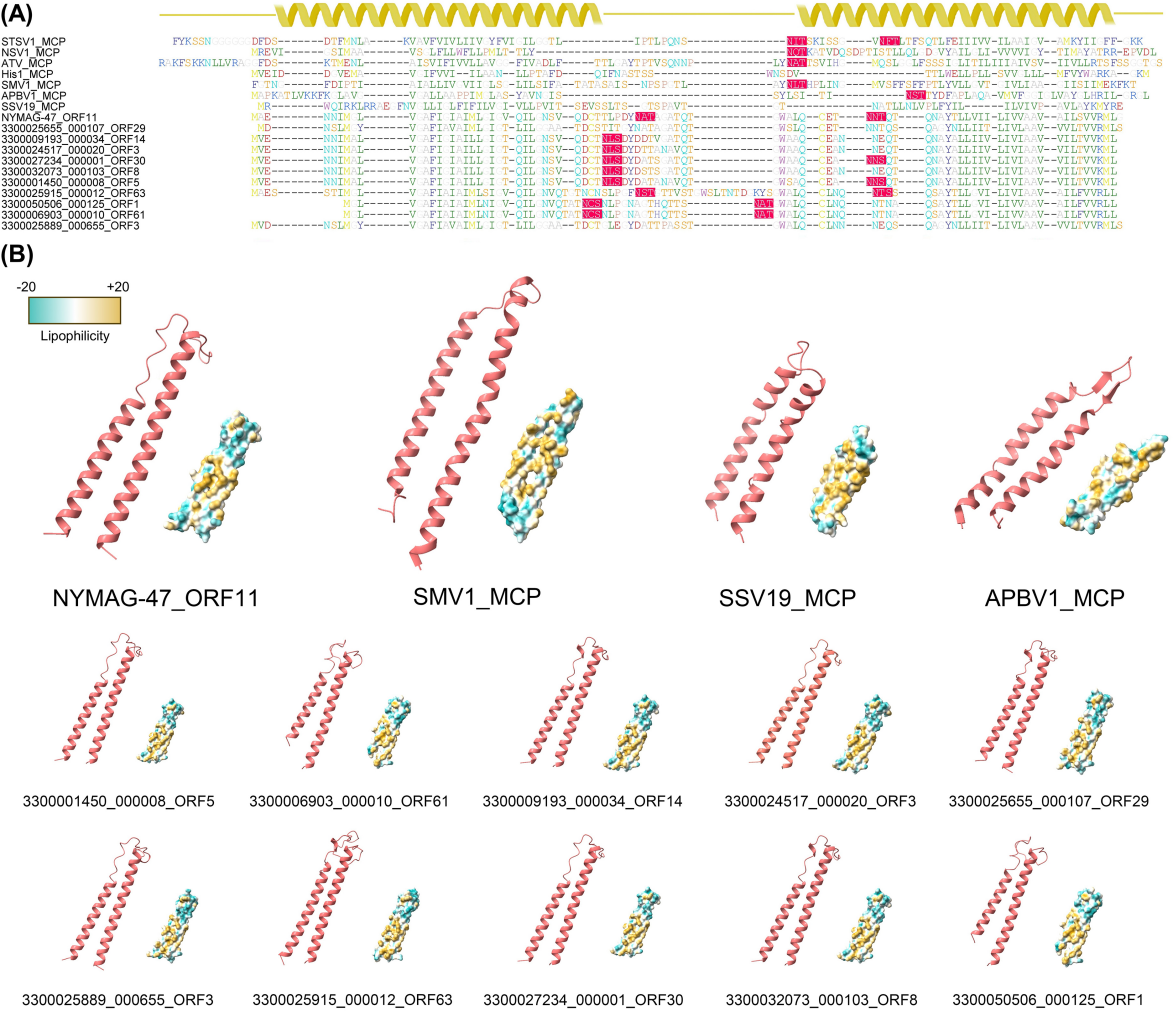
The MCP sequences and structures of NYMAG‐47 and related MCPs. (**A**) The sequence alignment between the MCPs of this study and the representatives of known spindle‐shaped viruses. Suspected *N*‐glycosylation sites are highlighted in red. The secondary structure is shown by the ribbons to represent the two hydrophobic α‐helixes predicted. (**B**) Predicted protein structures and lipophilicity profiles of NYMAG‐47, three spindle‐shaped viruses studied previously at high resolution, and 10 relatives of NYMAG‐47. The two α‐helixes are as shown in the ribbon model, and the sphere model indicates the lipophilicity.

The alignment of the genomes revealed multiple sets of homologous proteins shared amongst NYMAG‐47 and its 10 relatives (Figure 1), and are considered conserved within these genomes. One of the homologous proteins with a length of 89 amino acid residues in NYMAG‐47 showed a remote homology to that of the *Sulfolobus* spindle‐shaped virus 19 (SSV19) VP1, the MCP, during the HHpred and HMM searches with limited confidence (probability 75.4%). Subsequently, the structure of the suspected MCP of NYMAG‐47 was predicted and compared to the cryo‐EM structures of the SSV19 VP1, SMV1 MCP, and APBV1 MCP, resulting in significant matches with Z scores of 5.9, 4.3, and 3.5, respectively. These comparisons revealed that the suspected MCP of NYMAG‐47 shared a curved α‐harpin structure similar to that of the SSV19 VP1 and MCPs of SMV1 and APBV1 (Figure 2).

**FIGURE 3:**
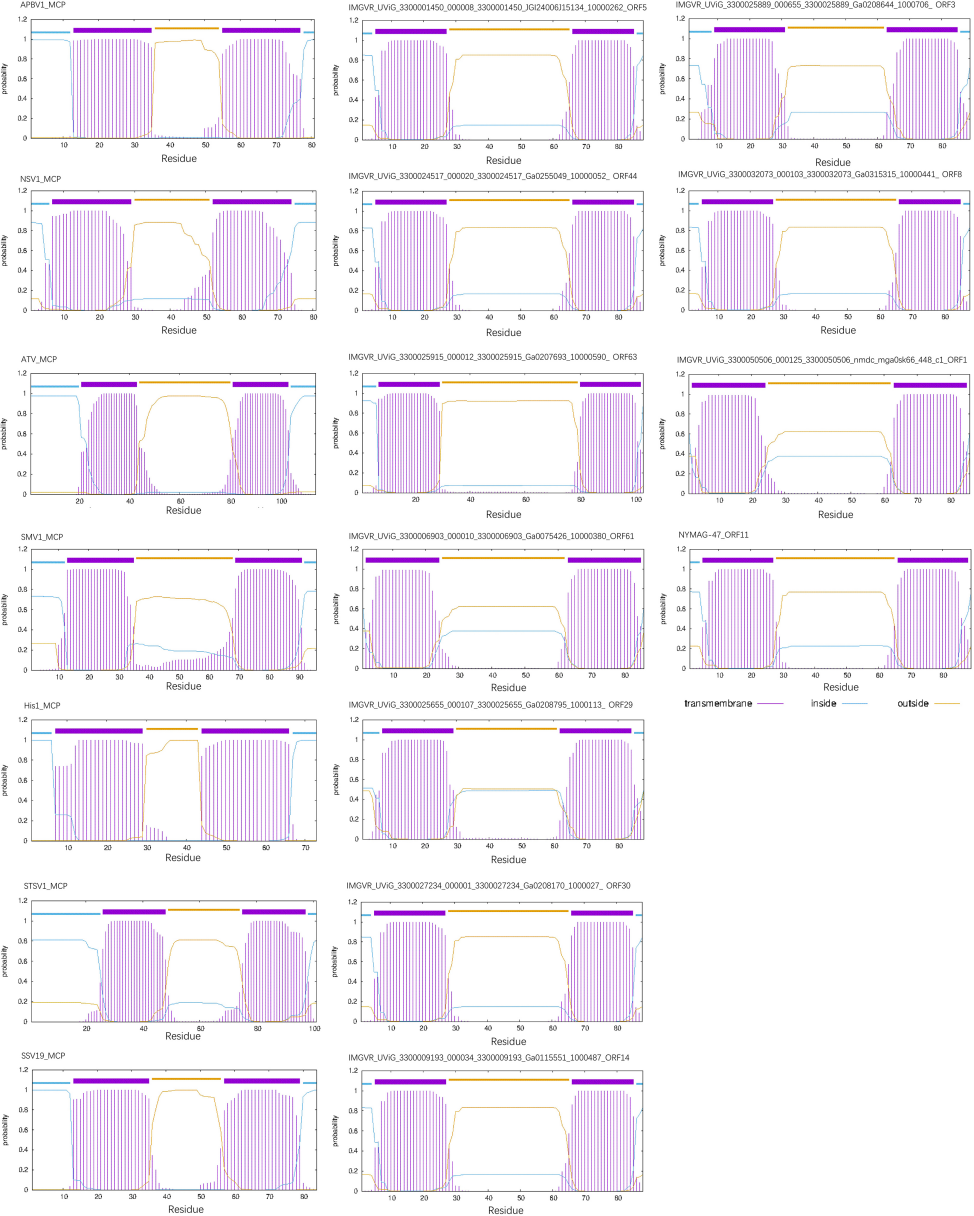
Transmembrane helix prediction of the deduced major capsid proteins of NYMAG‐47 recruited relatives, and representatives for known spindle‐shaped viruses.

Moreover, the predicted MCPs of NYMAG‐47 and its 10 relatives were found to have 0–2 potential glycosylation sites located on the turn of the hairpin (Figure 2). One of these sites was situated at the beginning of the second α‐helix, resembling the glycosylation site of STSV1 and APBV1, while the other site was located on the turn, unlike any previously observed MCPs (Figure 2). After virion formation, the turn of the helixes where these possible glycosylation sites locate was speculated to be on the outside of the capsid, as described previously (Wang et al., 2022), with potential functions yet unknown. The predicted MCP ‘outer‐membrane’ sections, the turn of the α‐helix hairpin of NYMAG‐47 and 10 close relatives, were longer than that of the previously discovered spindle‐shaped viruses by 10–20 residues (Figure 3), suggesting a possibly similar yet divergent outer‐capsid structure.

**FIGURE 4:**
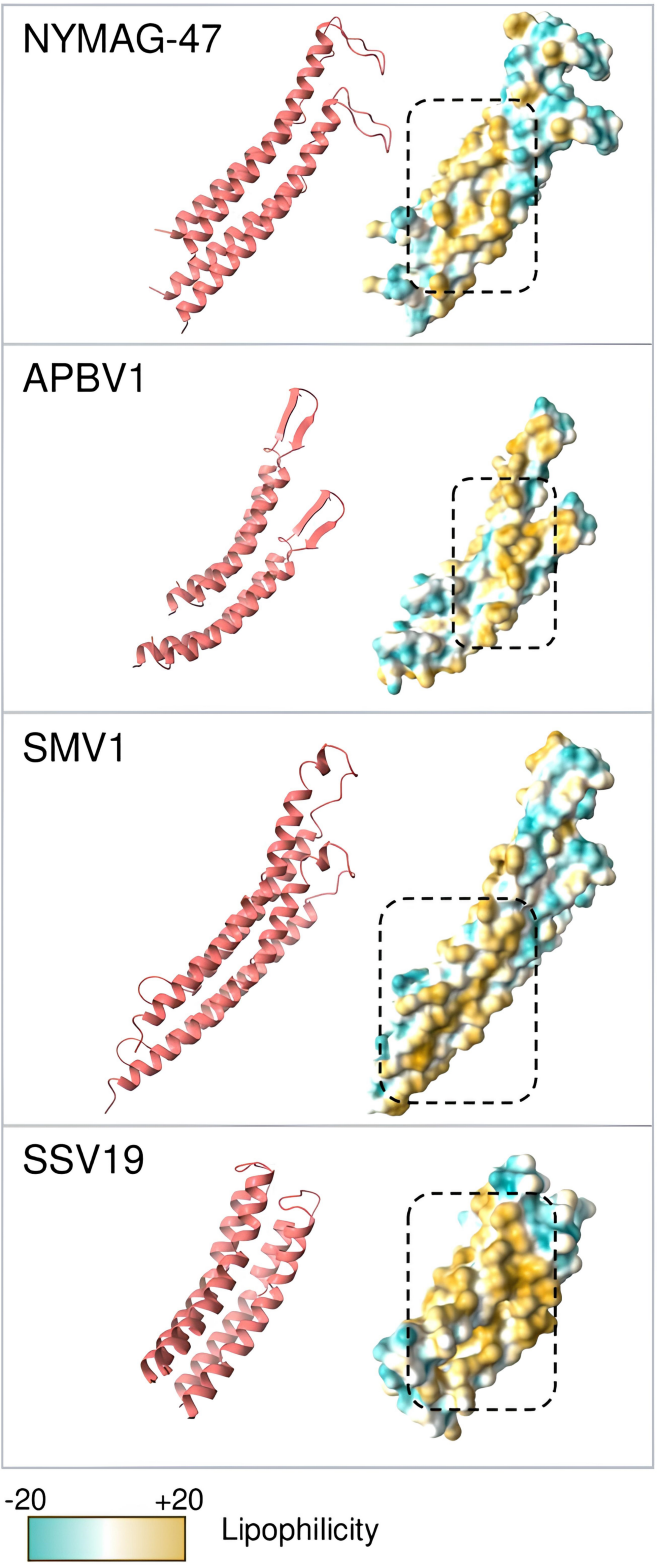
MCP docking prediction of NYMAG‐47 and 3 known spindle‐shaped viruses presented in ribbon model and sphere model. The hydrophobic interaction surfaces are framed with dashed boxes.

For further confirmation of a similarly assembled structure, a docking attempt was performed on the MCPs of NYMAG‐47 and three spindle‐shaped viruses at high resolution for a recreation of the actual interaction. The results showed that, in comparison to the known spindle‐shaped viruses, NYMAG‐47 received a high confidence score and Root Mean Square Deviation (RMSD) score (Table 1), indicating its potential for being assembled with the same conformation. The hydrophobic interfaces were found in the midsection of the MCP, enabling the joining of two MCPs and successively forming a chain (Figure 4). In contrast to the hydrophobicity, the two ends of the connected MCPs both showed a hydrophilic property, consistent with previous findings (Wang et al., 2022).

**TABLE 1:** MCP docking statistics of NYMAG‐47 and three known spindle‐shaped viruses.

	NYMAG‐47	APBV1	SMV1	SSV19
Score	−324.03	−269.4	−601.24	−311.35
RMSD (Å)	16.24	19.05	53.66	12.21
Confidence	0.9701	0.9159	0.9999	0.9618

Abbreviations: APBV1, *Aeropyrum pernix* bacilliform virus 1; MCP, major capsid protein; SMV1, *Sulfolobus* monocaudavirus 1; SSV19, *Sulfolobus* spindle‐shaped virus 19 (SSV19).

Notably, when conducting a structural comparison between all the proteins located at the tail of SSV19 (VP4, C131, and B210) and all of the modelled conserved proteins of NYMAG‐47, as well as the only three proteins with more than 500 residues, no significant structural similarity was observed. This suggests the presence of a different tail protein structure in NYMAG‐47. Additionally, no complete endo‐mannanase‐like motif was identified in the proteins of NYMAG‐47 and its related strains, further supporting the hypothesis of a potentially novel tail structure.

Collectively, the findings from the analysis of MCP, such as structural similarity, the overall *N*‐glycosylation site on the turn of the hairpin, the hydrophilicity profile, and the docking result (Figures 2, 3, 4), support the interactions between the putative MCPs and the potential glycosylation modifications of NYMAG‐47. These features resemble those observed in rod‐ to spindle‐shaped viral MCPs (Quemin et al., 2015; Wang et al., 2022). Thus, it is speculated that these viruses are morphologically unrelated to *Caudoviricetes*, but rather to the spindle‐shaped ones.

There was no available annotation with high confidence for glycosyltransferases found on NYMAG‐47, and for all but one relative recruited, multiple available *N*‐glycosylation sites were found. Therefore, either genes of the suspected function were too novel to be identified, or NYMAG‐47 relies on its host for such a process, like that of SSV1 (Quemin et al., 2015) but unlike the genes encoded directly on the genomes of His1 and NSV1. This is fascinating since its putative host is a mesophilic organism, yet it was deduced that viral capsid glycosylation, or membrane protein glycosylation in general, could serve as protection (Wang et al., 2022). Viral capsid glycosylation is a highly diverse modification for many purposes, including self‐supporting like capsid stabilization for the virion (Khayat et al., 2010), proliferation strategies like host recognition (Hartman et al., 2020), and escaping the immune response (Feng et al., 2022). Thus, it is hypothesized that for the glycosylation of these virions found in this study and some other similar virions (Pietilä et al., 2013), their glycosylation properties could serve as a customized modification for host adhesion like that of the HRPV‐1 (Kandiba et al., 2012). The adhesion mode of SMV1 supports this hypothesis (Uldahl et al., 2016) to some extent by having the entire capsid flattened and attached to the host membrane instead of just the tail, like in NSV1 (Kim et al., 2019).

The identification of MCP of these genomes only through structural prediction instead of sequence comparison explains to a certain extent why new spindle‐shaped viruses were hardly identified in metagenomic datasets, while they are ubiquitous in environments (Krupovic et al., 2014). This could also suggest that the short, simple‐structured proteins of the same function could pack a greater variation in their sequence by allowing residues of similar conformation to replace the previous ones, providing greater evolutionary diversity, or being a result of convergent evolution. All of the studied spindle‐shaped virus MCPs with electron microscopy images of the relatively higher resolution were found to form a helical unit as the subunit and assemble the capsid with a seven‐fold rotational symmetry (Han et al., 2022; Wang et al., 2022). With the MCPs discovered in this study and the assembled structure previously studied, the possibility of these viruses being rod‐shaped was also considered, though their genome sizes (27,181–38,019 bp) suggest that it is more likely to be a spindle‐shaped one with an even larger virion than the NSV1 (27,548 bp) given the positive correlation of genome size and capsid volume (Wang et al., 2022).

### Host of NYMAG‐47 and its 10 relatives

NYMAG‐47 was predicted to be an archaeal virus by iPHoP, which aggregates the host prediction results from BLAST, CRISPR, WIsH, VHM, PHP, and RaFAH through machine learning means (Ahlgren et al., 2017; Coutinho et al., 2021; Galiez et al., 2017; Johnson et al., 2008; Lu et al., 2021; Roux et al., 2023). Specifically, it was predicted to be the virus of archaea within the genus *Nitrosopumilaceae* TA‐20 and some of the other genera within the family *Nitrosopumilaceae*, for example, *Nitrotenuis*, *Nitrosoarchaeum*, and *Nitrosotalea*, though with limited confidence (0.55–0.67). Given the insufficient credibility of such a prediction, we also inspected the similarities in proteins. Fourteen proteins of NYMAG‐47 shared a significant identity (coverage 53%–100%; identity 32.6%–77.2%) to that of the archaeal members within *Nitrosopumilaceae* and one protein to an AOA virus (NSV1) in the non‐redundant protein database (Table S1). The 10 relatives of NYMAG‐47 also shared many suspected homologous genes (112 proteins in total, coverage 50%–100%; identity 31.3%–85.2%) with *Nitrosopumilaceae* members (*Nitrosopumilaceae* archaeon, *Nitrososphaerota*, *Candidatus* Nitrosopelagicus, *Nitrosopumilus*) and one with NSV1 (Table S1).

Genetic homology can be used to deduce virus‐host correlation (Edwards et al., 2016), but it is unusual for a virus to have proteins of such quantity to possess homology to its host. Subsequently, CheckV was applied to the original contigs of the BLASTp hits collected from GenBank (*n* = 38, sequence length mean = 21,838 ± 40,794 bp) (Table S4), which indicates that eight contigs collected possess viral proteins, 15 contigs contain neither detectable viral nor host proteins, and eight contigs contain more than 10 proteins of host origins (*Candidatus* Nitrosarchaeum limnium, Marine Group I Thaumarchaeota, *Nitrosopumilus* sp., *Nitrosopumilaceae* archaeon, and other archaea). This suggests that amongst these contigs, not all were of definitive archaeal origin, especially when 31 of 38 contigs were binned metagenomic‐assembled genomes. Other than hypothetical proteins, no other proteins were found with annotations in BLASTp hits against contigs containing more than 10 proteins of host origin. Upon inspection after filtering out the possibly mis‐binned contigs, NYMAG‐47 had a methyltransferase (NYMAG‐47_15) sharing high identity (coverage: 97%; identity: 77%) with what was encoded on a *Candidatus* Nitrosarchaeum limnium SFB1 contig. The other 10 virus genomes each encoded 2.5 ± 0.8 proteins sharing significant similarity with proteins of confident AOA origin (RefSeq single protein deposit, or contigs possessing 10 or more host genes) on average.

**FIGURE 5:**
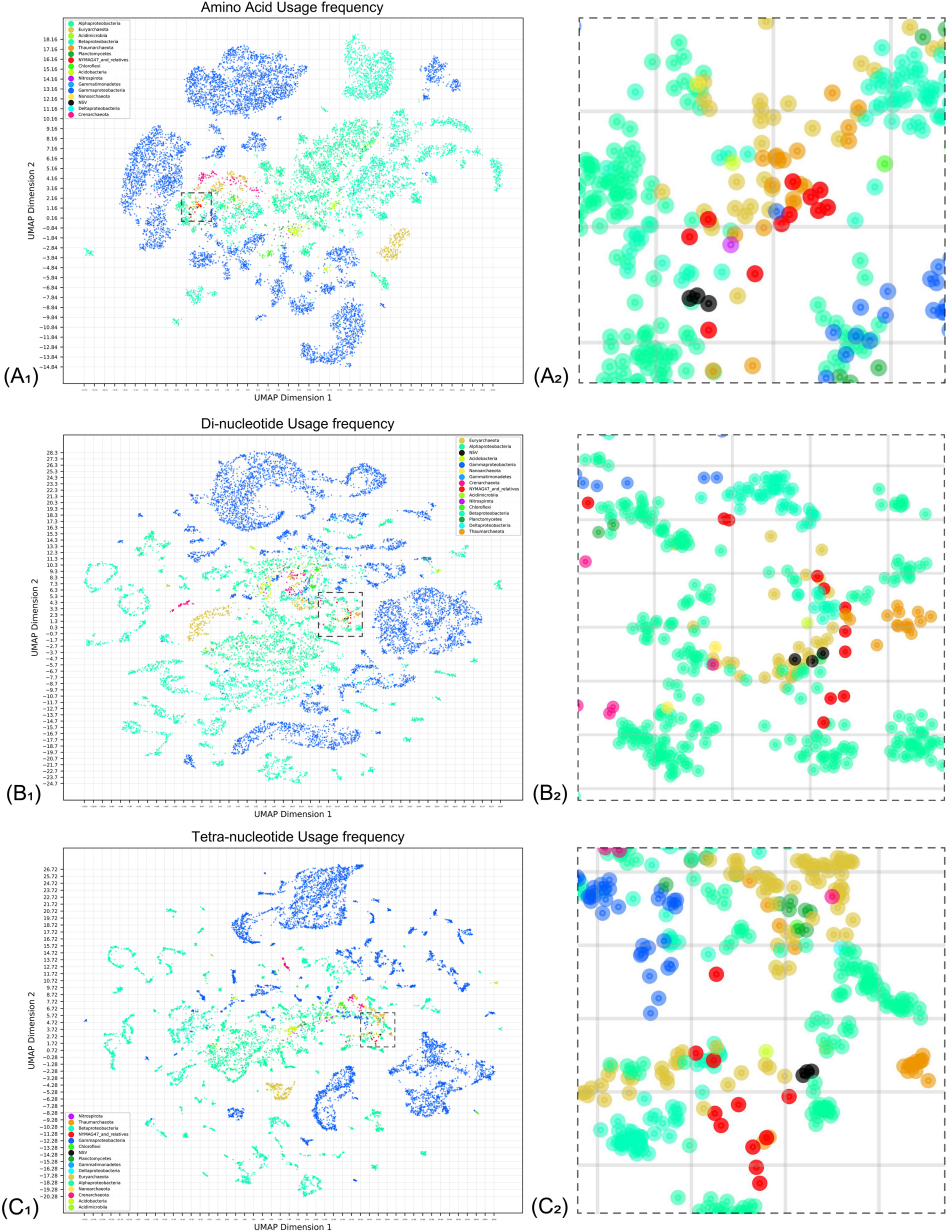
The visualized UMAP dimensionality reduction maps of amino acid usage, di‐ and tetra‐nucleotide frequency of NYMAG‐47 and other organisms. Each dot represents a genomic sequence. Different organisms are indicated in different colours. NYMAG‐47 and its 10 relatives are coloured in red (A_1_, B_1_, C_1_). Global visualization of the usage frequencies, including Proteobacteria, Acidobacteria, Acidimicrobiia, Planctomycetes, Gemmatimonadetes, Nitrospirota, Acidimicrobiia, Chloroflexi, Euryarchaeota, Nanoarchaeota, and Crenarchaeota, NSV1‐3, and NYMAG‐47 and its relatives. Regions including NYMAG‐47 and related genomes are marked using a dashed line. (A_2_, B_2_, C_2_) Enlargement of NYMAG‐47 and related genomes in (A_1_, B_1_, C_1_).

To further support such a claim of possibly mis‐binning, the amino acid usage frequency (AAUF) and the di‐/tetra‐nucleotide usage frequency (D/TUF) of major microbial players in Yangshan Harbour (Wang et al., 2015; Zhou et al., 2023) were analysed and visualized alongside with that of NYMAG‐47 and related viruses. As shown in Figure 5, NYMAG‐47 and its relatives exhibited higher similarities of AAUF and D/TUF to AOA of Thaumarchaeota and certain Euryarchaeota compared to most of the predominant bacterial groups. This finding potentially explains to what end these related viral contigs were previously binned with genuine AOA contigs, and provides additional correlation to this hypothetical virus–host match.

The possibility of NYMAG‐47 having *Nitrosopumilaceae* as its host is also supported by our previous discovery that 84% of the archaeal community in Yangshan Harbour was made up of the *Nitrososphaeria* archaea (Zhou et al., 2023), representing the AOA community. It is worth noticing that, at the time of writing, no bacterial spindle‐shaped viruses had been identified (Krupovic et al., 2014), and no AOA were confirmed to have exosomes. The recruited environmental genomes (10 relatives of NYMAG‐47) were originally collected from various metagenomic data of inlets, coastal areas, intertidal zones, epipelagic and oceanic waters, and even soil, matching the typical distribution profile of AOA (Kim et al., 2021) (Table S2). They are not local‐only viruses and have yet to be recognized due to their genomic novelty. Currently, our attempts at isolating AOA from the Yangshan harbour water sample have made promising progress (unpublished data), and the isolation of the AOA viruses from this aquatic environment is expected to be approached.

Sequence match of neither tRNA genes nor CRISPR spacer–protospacer was identified between these viral genomes and prokaryotic genomic sequences available publicly.

### Classification of NYMAG‐47 and its 10 relatives

Albeit putatively, the host from the same family and even the similar virion morphology, NYMAG‐47 had little sequence similarity to the known NSVs, with most of its gene functions unable to be annotated with conventional database comparison, including the MCP, which explains why it was not previously identified (Zhou et al., 2023). Remote homology searches with HHblitz also annotated 52 proteins of NYMAG‐47 to be of unknown functions, with three of them present in the conserved gene cluster shared amongst all relatives (Figure 1). ATPase found on these genomes (Figure 1) was shared as a putative core gene, and it was detected in all spindle‐shaped viruses previously discovered, correlating to genome replication (Krupovic et al., 2014). The family B DNA polymerase of His1 and NSV1 was not detected on the genome of NYMAG‐47, suggesting a different set of conserved genes amongst its lineage.

**FIGURE 6:**
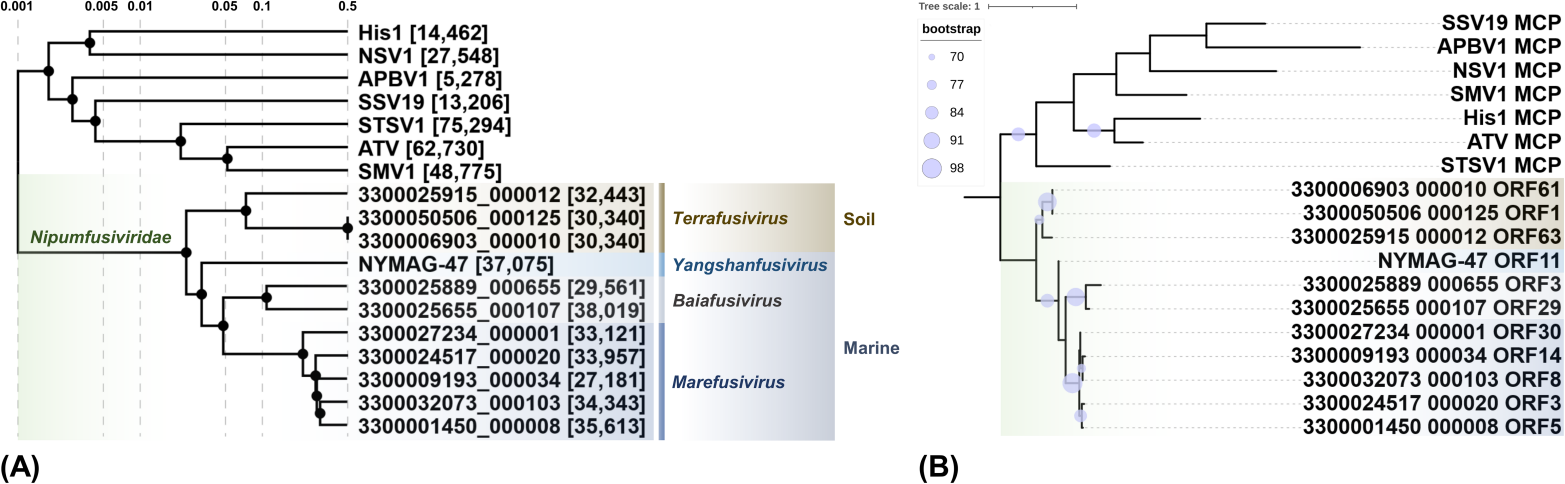
The phylogeny of NYMAG‐47 recruited relatives and known spindle‐shaped archaeal viruses. The proposed four new genera are shown in different colours at the end of the branches. (**A**) Whole genome proteomic tree. The S_g_ value is labelled on the top, and the proposed taxonomy and sequenced sample origin of NYMAG‐47 and its relatives are included. Lengths of each virus genome are shown in the square bracket (bp). (**B**) Major capsid protein tree. Only nodes with bootstrap values higher than 70% are shown.

The phylogenetic trees of MCPs and the whole‐genome‐based proteomes both revealed a distinctly different lineage of NYMAG‐47 and its 10 relatives in comparison to the known spindle‐shaped archaeal viruses (Figure 6). It suggests that these viruses could be classified into their distinct clade of possibly similar morphology. The phylogenetic affiliations of NYMAG‐47 and 10 relatives remained almost identical in this clade (Figure 6). This consistency serves as additional confirmation of the conservative nature of the viral properties and the inferred accuracy of the putative MCPs.

Based on the S_g_ value and the presence of a monophyletic branch alongside a shared set of core genes, NYMAG‐47 and its 10 relatives could be classified into four different genera within the same family (Figures 1 and 6). We propose a new family for NYMAG‐47 and its relatives (Table S2), the *Nipumfusiviridae* (‘Ni’ and ‘pum’ for having sequence features similar to archaea from the family *Nitrosopumilaceae* and for being the deduced host; ‘fusi’ after the Latin word meaning spindles for the possible morphology). The four genera are named *Yangshanfusivirus*, *Terrafusivirus*, *Marefusivirus*, and *Baiafusivirus* after their original sampling sites, and species names are based on the sampling locations (Table S2). To be classified within this proposed family, it is recommended that the new members should share a set of conserved genes, including the protein with a Zinc‐binding domain, three proteins with yet unknown functions, the MCP, and the ATPase.

## CONCLUSION

The genome of a putative *Nitrosopumilaceae* spindle‐shaped virus was identified in Yangshan Harbour, the East China Sea. The annotation of its MCP was achieved through structural comparison, *N*‐glycosylation site recognition, hydrophilicity prediction, transmembrane helices prediction, and docking simulation. Its 10 close relatives were recruited from geographically diverse locations of iconic ammonia‐oxidizing archaea distribution, representing a new branch of spindle‐shaped archaeal viruses. Our findings shed light on the diversity as well as potential ecological roles of AOA viruses in mesophilic environments.

## AUTHOR CONTRIBUTIONS

**Yimin Ni:** Conceptualization (supporting); data curation (equal); formal analysis (lead); investigation (lead); methodology (lead); validation (equal); visualization (equal); writing – original draft (lead); writing – review and editing (equal). **Tianqi Xu:** Methodology (supporting); software (equal); visualization (equal). **Shuling Yan:** Conceptualization (equal); resources (supporting). **Lanming Chen:** Conceptualization (supporting); investigation (supporting); resources (supporting). **Yongjie Wang:** Conceptualization (lead); data curation (equal); formal analysis (supporting); funding acquisition (lead); investigation (supporting); methodology (supporting); project administration (lead); resources (supporting); supervision (lead); validation (supporting); visualization (supporting); writing – original draft (supporting); writing – review and editing (lead).

## CONFLICT OF INTEREST STATEMENT

The authors declare no conflicts of interest.

## Supporting information


**TABLE S1.** Original BLASTp data of viral genomes of this study sharing similarities to archaeal proteins. Only the top hit of each match is shown, and hits sharing similarities with known ammonia‐oxidizing archaeal viruses are also kept for reference. Hits made to contigs including more than 10 proteins of host origin or RefSeq proteins are highlighted in red.
**TABLE S2.** General information of NYMAG‐47 and recruited relatives, including G + C content, genome length, original sampling site, and original study name of the recruited genomes. Proposed taxonomic names are also included.
**TABLE S3.** Collection of annotations of NYMAG‐47 proteins including coordinates and predicted functions of each protein.
**TABLE S4.** CheckV results of original sequences deposited in GenBank sharing protein similarity to genomes in this study during BLASTp alignment.
**TABLE S5.** Dot coordinates of genomes downloaded from GenBank, NYMAG‐47, and related genomes during UMAP visualization for amino acid usage frequency (Figure 5A).
**TABLE S6.** Dot coordinates of genomes downloaded from GenBank, NYMAG‐47, and related genomes during UMAP visualization for di‐nucleotide frequency (Figure 5B).
**TABLE S7.** Dot coordinates of genomes downloaded from GenBank, NYMAG‐47, and related genomes during UMAP visualization for tetra‐nucleotide frequency (Figure 5C).

**FILE S1.** Complete genome of NYMAG‐47 and the recruited members discussed in this study in fasta format.

## Data Availability

The full genome of NYMAG‐47 was uploaded to GenBank under accession number OR367329, the raw genomes of recruited relatives can be accessed through https://img.jgi.doe.gov/cgi-bin/vr/main.cgi with the IDs included in Table S2. The clean cyclized genome can be accessed from File S1 directly.
